# Long-term psychobiological stress responses following soft political repression

**DOI:** 10.1038/s41598-026-65049-8

**Published:** 2026-08-06

**Authors:** Ruth Marheinecke, Laura Ramirez, Nils Opel, Carsten Spitzer, Bernhard Strauß, Veronika Engert

**Affiliations:** 1 Institute for Psychosocial Medicine, Psychotherapy and Psychooncology, Jena University Hospital, Friedrich-Schiller University, Stoystraße 3, 07743 Jena, Germany; 2 Department of Psychiatry & Neuroscience, Campus Benjamin Franklin, Charité Universitätsmedizin Berlin, Berlin, Germany; 3 Department of Psychosomatic Medicine and Psychotherapy, University Medical Center Rostock, Rostock, Germany; 4 German Center for Mental Health (DZPG), Berlin, Germany; 5 Center for Intervention and Research in adaptive and maladaptive brain Circuits underlying mental health (C-I-R-C), Halle-Jena-Magdeburg, Jena, Germany

**Keywords:** Neuroscience, Psychology, Psychology

## Abstract

The sociopolitical environment can act as a potent stressor with significant consequences for health and well-being. Of increasing global relevance in this context is soft political repression, including denunciation and surveillance, which is employed to prevent oppositional behavior while minimizing international attention. Although common in authoritarian regimes, including the former German Democratic Republic (GDR; 1949–1990), little is known about its psychobiological health consequences. We examined 100 individuals raised in the GDR (49 exposed to repression, 51 matched controls) to investigate stress responses, a key pathway to long-term health. Participants completed questionnaires and, if health-related inclusion criteria were met and they felt able to complete the task, underwent the Trier Social Stress Test (TSST; repression *n* = 29, controls *n* = 46). Subjective stress, cortisol, heart rate, and heart rate variability were assessed. The repression group reported higher stress, but did not differ from controls in physiological measures. Subgroup analyses showed that repression participants who declined attending the TSST exhibited elevated symptoms of distress, anxiety, depression and trauma, whereas completers were comparable to controls. Our findings suggest differential long-term consequences of soft political repression, with preliminary evidence for both vulnerable and resilient groups and raise broader methodological questions regarding the ecological validity of laboratory-based stress research in vulnerable groups.

**Supplementary Information:**

The online version contains supplementary material available at 10.1038/s41598-026-65049-8.

## Introduction

In recent years, awareness has grown that the broader socioecological environment, including sociopolitical uncertainty and societal instability, can act as potent stressors with long-term consequences for health and well-being (such as living conditions in ongoing armed conflict^1^, sociopolitical uncertainty in Lebanon^2^, or climate change^3^. One stressor of growing global relevance is soft political repression, a covert form of state-organized psychosocial pressure that aims to intimidate, destabilize, or socially isolate targeted individuals to prevent them from engaging in oppositional behavior, while minimizing international attention and accountability (for recent review of categorizations of repression including a broader set of actors and targets, see^4^) ^5–8^. Soft repression constitutes a central instrument of power in authoritarian regimes, but can also be present in democratic or hybrid regimes, including practices such as surveillance, limitations on freedom of speech, or the intentional creation of difficulties in individuals’ private and professional lives^9–11^. The 2025 Freedom in the World Report depicts a current rise in authoritarianism accompanied by a decline in democratic freedom, suggesting a global increase in soft repression practices^12^. Little is known about the psychological and behavioral consequences of soft repression for affected citizens, and even less about its physiological impact. Evidence from diverse geopolitical contexts, including Hong Kong^13^, Zimbabwe^14^, Colombia^15^, Venezuela^16^, Thailand^17^ or Sweden^18^, suggests that soft repression can be associated with psychological outcomes such as fear and depressive states, as well as behavioral effects including self-policing, political retreat or migration. A major obstacle to studying these processes is the difficulty of reaching individuals who are currently exposed to repression, as restrictive conditions severely limit access and participation. Furthermore, fear of reprisals can lead to selective non-response or overly cautious reporting. One way to address these challenges is to draw on historical samples still accessible today.

A historically well-documented example of soft political repression is a set of techniques used by the Ministry for State Security (MfS) in the former authoritarian German Democratic Republic (GDR; 1949–1990) known as “Zersetzung” (engl: disintegration). In a recent study, around 10% of former GDR citizens report having been subjected to soft repression during the existence of the GDR^19^. Repression measures included persistent overt and covert surveillance by the Ministry for State Security (known as *Stasi*, the GDR’s secret police), harassment, humiliation, or exclusion in educational and workplace settings, as well as the deliberate damaging of someone’s reputation, by for example spreading rumors or placing an informant in the target’s close circles:

*“I submitted an application to leave the GDR. And from that moment on*,* I was NEVER alone anymore. I always had the feeling that someone was with me. No matter where I parked my car*,* there was always another car there with me (…) And I didn’t know then that my children would have to suffer because of it. After the application*,* they had to endure constant harassment at school (…) One time on a trip to Berlin*,* a Stasi car stopped next to us*,* pulled us inside*,* and questioned each of us separately about what we wanted*,* accusing us of planning to flee.” (Anonymous Participant of current study; shortened and translated quote).*

As the above quote from an affected individual illustrates, soft repression was characterized by constant feelings of insecurity and anticipatory anxiety, reflecting a prolonged state of perceived threat and chronic psychosocial stress, which has repeatedly been linked to adverse health outcomes^5,20,21^. Accordingly, in a study conducted in 1994, individuals affected by soft repression in the GDR reported non-specific somatic symptoms such as agitation, restlessness, sleep disturbances, and excessive sweating, indicative of a prolonged stress response^22^. Furthermore, they showed high prevalence rates of recurrent depression, post-traumatic stress disorder (PTSD), somatoform disorders, and anxiety disorders. Later studies have suggested that these mental health consequences persist decades after GDR repression and can approximate those observed among former political prisoners, including an increased prevalence of affective and anxiety disorders^5,22–24^. Despite this substantial psychological burden, individuals exposed to soft repression often face barriers to official recognition, including limited access to rehabilitation and compensation, as well as persistent stigma, e.g. in healthcare settings^25,26^. Consistent with this, a recent study found that German healthcare professionals held fewer positive and more negative attitudes toward individuals with a history of political repression than toward those with a general GDR background^26^, leaving many affected individuals feeling unseen and misunderstood^5,25^. As soft repression is designed to induce uncontrollability, unpredictability and social isolation, core factors of chronic stress associated with neuroendocrine and immunological responses^27^, stress physiology provides a theoretical framework for understanding its health consequences.

Stress is defined as the organism’s response to perceived threats to homeostasis^28^. When faced with a stressor, acute activation of the sympathetic adrenal medullary (SAM) system causes the release of epinephrine, which induces rapid physiological changes including increased heart and respiration rates, preparing the organism for “fight-or-flight” responses. Activation of the slower hypothalamic–pituitary–adrenal (HPA) axis leads to the secretion of cortisol, a glucocorticoid hormone that mobilizes energy resources and modulates immune functioning^29^. While the acute stress response is adaptive, persistent and uncontrollable stressors, especially those threatening the social self, can lead to chronic dysregulation of stress system (e.g., hypocortisolism) and immune system and an elevated risk for mental and physical illness, as demonstrated in studies on severe stressors such as bullying and discrimination^27,30–34^. At the same time, not all individuals exposed to chronic stress continue to develop mental or physical health problems. Research has identified several mediating and moderating variables, including social support, resilience, and contextual factors such as socioeconomic status, as important in shaping these outcomes^21^.

In an article investigating the same sample as included here, we show that more than 30 years later, experience of soft repression in the GDR was associated with higher symptoms of anxiety, depression and trauma symptoms, higher systemic inflammation and shortened telomere length, the latter conditional to low social support^6^. Building on this foundation, and focusing on the underlying physiological mechanisms, the current study investigates whether individuals exposed to soft political repression exhibit measurable alterations in psychobiological stress responses. Specifically, and reflecting a state of HPA axis downregulation after decades of elevated stress levels, we hypothesized that victims of repression compared to a control group would exhibit blunted cortisol reactivity in response to a standardized psychosocial stressor, the Trier Social Stress Test (TSST), widely known as the gold standard for psychosocial stress induction^35^. We further expected them to show elevated subjective stress and sympathetic activity (heart rate) as well as decreased parasympathetic activity (heart rate variability). Moreover, given their known relevance to health outcomes, we expected socioeconomic status (SES), resilience, and social support to act as pathways from repression experiences to altered stress responses^6,21^. For feasibility reasons, and deviating from our original preregistration, we treated these factors as moderators, examining if they attenuated the associations between repression experience and acute stress response. In an exploratory analysis, we additionally examined psychological differences between individuals capable and willing to participate in the stress test and those who refused or who needed to be excluded for health reasons.

## Materials and methods

### Study design

This cross-sectional study was conducted in Germany, with participant recruitment and data collection carried out between June 2022 and June 2024. The study’s overall hypotheses and methods were preregistered at OSF, after data collection but before accessing any data due to logistical considerations (https://osf.io/s2c6p/overview). Not all pre-registered variables were included in this manuscript, as they are either reported elsewhere^6^ or being prepared in a separate publication. In manuscript preparation, STROBE guidelines were used. The authors assert that all procedures contributing to this work comply with the ethical standards of the relevant national and institutional committees on human experimentation and with the Helsinki Declaration of 1975, as revised in 2013. This study was approved by the ethics committee of Jena University Hospital (No. 2022–2605_1-BO).

### Participants

The recruited sample consisted of 100 former citizens of the GDR, aged between 50 and 78 years, predominantly from the states of Thuringia and Saxony in Germany. Participants were recruited through a combination of distributed flyers, advertisements in local newspapers, and collaborations with community or religious organizations. Two independent groups were recruited: For the repression group, outreach specifically addressed individuals who had experienced soft repression in the GDR, with examples provided in recruitment materials. The control group was recruited more broadly, targeting former GDR citizens aged 50 and older. During recruitment, both groups were matched for age, gender, and regional origin. Final group assignment and eligibility were assessed via structured telephone interviews. In the repression group (*n* = 49), participants reported at least two experiences of soft repression, such as systematic surveillance, denunciation or targeted harassment in professional or educational contexts. The control group (*n* = 51) did not report personal experiences of soft repression.

Exclusion criteria for both groups were (1) political imprisonment at any time, (2) current use of medications affecting cortisol release (e.g., steroids, antidepressants), (3) current clinical diagnosis of an affective disorder or PTSD within the past two months, or exceeding cut-off values for clinical depression in the telephone screening (4) psychotic symptoms within the past two years, (5) excessive use of alcohol or recreational drugs, and (6) only for the TSST: any severe physical health impairment that could be triggered in the acute stress setting (e.g. asthma, cardiovascular disease). In addition to these initial researcher-imposed exclusion criteria, eligible participants were still free to decide whether or not to take part in the TSST. Participants who were unable to attend the TSST for health-related reasons or who chose not to participate could nevertheless take part in the other study parts. Participants received a monetary compensation of 10€/hour for participation, resulting in a maximum of 70€ for participants of the repression group and 55€ for participants of the control group.^6,36^.

### Procedure

**Fig. 1:**
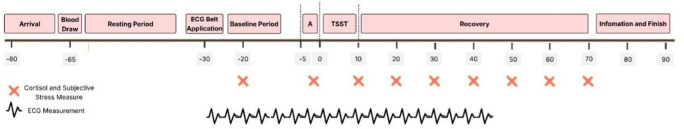
Testing Timeline. A = Anticipation Phase, TSST = Trier Social Stress Test. This Figure was created using lucidchart.com.

Upon first interest, a structured telephone interview was conducted to evaluate inclusion and exclusion criteria and to allocate participants to repression or control groups. During this call, participants were also informed about the nature of the TSST. Several individuals, particularly from the repression group, were excluded from or opted out of the TSST at this point, yet chose to participate in the other study components. As a result, sample sizes varied across different study sections (see Fig. 1). Eligible participants subsequently completed a questionnaire battery. The questionnaire battery included demographic information (e.g., income, education, relationship status, sex, age) and standardized psychological measures for constructs such as resilience, social support, depression, anxiety or PTSD symptoms. Following completion of the questionnaires, participants were scheduled for a late morning laboratory session to take part in the TSST – the gold standard procedure to reliably induce subjective and physiological stress responses in the laboratory setting^35,37^. TSST sessions were scheduled in the late mornings rather than in the afternoon as is typically recommended. This decision was a compromise that allowed integration of other study components requiring morning blood sampling, while nevertheless placing the TSST in a time window after the expected peak of the cortisol awakening response^38^. Importantly, participants spent approximately 60 min in the laboratory before collection of the first saliva sample (see Fig. 1), which likely further reduced any immediate influence of the cortisol awakening response on basal cortisol levels. All participants provided written informed consent.

#### Laboratory procedure

We instructed participants to not eat, drink caffeinated beverages, smoke, or engage in physical activity for at least two hours prior to testing. Sessions were rescheduled if participants reported symptoms indicative of acute infection. Participants arrived at the laboratory between 9:30AM and 11AM. They were allowed time to settle in before being provided with an overview of the upcoming 2.5-hour testing procedure. As part of a separate study component, a blood sample was collected shortly after arrival. Following a 30-min resting period, participants were fitted with an ECG belt and a 15-minute baseline ECG recording was conducted. Participants were then introduced to the saliva sampling method using Salivettes (Sarstedt™) and to our acute subjective stress scales, the State-Trait Anxiety Inventory (STAI) and a 7-point Likert-scale asking “how stressed do you feel?”. After this, the first saliva sample and subjective stress assessment were collected. Participants were then guided into the adjacent TSST room, where two trained confederates were already present. The experimenter read the standardized TSST instructions aloud. After a 5-minute anticipation phase, the second salivary cortisol and subjective stress samples were collected, and immediately following the 10-minute TSST, the third samples were collected. Participants were then returned to their resting room, where six additional salivary cortisol and subjective stress measurements were collected at 10-minute intervals during the 60-minute recovery phase. The session concluded with a thorough debriefing.

#### Trier social stress test

For the TSST, participants underwent a 5-minute mock job interview, followed by a 5-minute mental arithmetic task in front of a committee of two confederate ‘behavioral analysts’. The confederates were trained not to engage with the stressed target outside of task instructions, not to provide any type of feedback, and to maintain a neutral demeanor (see S.1). To ensure the TSST was manageable for the vulnerable repression group, several adaptations to the original protocol^35^ were implemented. Cameras and microphones were removed and research assistants received a training with clear guidelines for recognizing and responding to severe psychological distress or signs of dissociation. Despite these precautions, it became evident that the TSST remained a highly intense stressor for some participants. Several repression group individuals reported that specific features of the testing environment, such as the bare, dark room, spatial confinement, use of face masks due to COVID-19 regulations, and the basement location of the laboratory, evoked strong associations with former secret service interrogation settings in the GDR. Therefore, after the testing of eight participants in each group, the stress testing procedure was relocated to a larger, well-lit room. This setting allowed for greater physical distance between participants and the TSST committee, contributing to an increased sense of safety. In addition, to enhance participants’ perceived control, information about the TSST was provided before the collection of the baseline Salivette, and committee members briefly introduced themselves before assuming their roles in the task. All modifications were implemented directly in response to participant feedback.

### Measures

#### Verification of group allocation: repression intensity

As a manipulation check of successful group allocation, participants were asked to report all types of repression they had experienced in the GDR (see Tables S.2). We compiled a sum score of repression experiences and compared repression and control groups on this indicator, expecting substantial between-groups differences.

#### Subjective stress and arousal

To assess general subjective life stress, we administered the German version of the 10 Item Perceived Stress Scale (PSS^39^. Subjective stress during the laboratory testing was assessed at nine time points (alongside cortisol measures) using the German version of the 20-item state scale of the State–Trait Anxiety Inventory (STAI^40^, a validated and widely used measure of subjective stress. Because the STAI primarily assessed anxiousness rather than stress per se, we additionally included a 7-point Likert-scale measure (“How stressed do you feel?”) and merged both scales into an equally weighted composite subjective stress score.

#### Salivary cortisol

As a measure of physiological stress, we collected salivary cortisol at nine time points during the laboratory testing, using Salivettes^®^ (Sarstedt). Samples were stored at -80 °C and sent in two batches (June 2023 and June 2024) to the Biochemical Laboratory of the Department of Biological and Clinical Psychology of Trier University for analysis. Cortisol levels (in nmol/L) were assessed using a time-resolved fluorescence immunoassay with intra-/interassay variabilities of < 10%/12%, identical for both batches^41^. All samples were assayed in duplicate, and mean values were used for statistical analyses.

#### Heart rate and heart rate variability

As measures of sympathetic and parasympathetic activity, heart rate (HR) and heart rate variability (HRV) were assessed using continuous electrocardiogram (ECG) recordings. Participants wore the Zephyr BioHarness 3 chest belt (Zephyr Technology, Annapolis, MD, USA), which continuously recorded ECG data at 250 Hz for a total of 75 min (-30 to + 45 min relative to stressor onset at 0 min). Sympathetic nervous system activity was indexed by heart rate, expressed in beats per minute (bpm). As HRV value, we used the Root Mean Square of Successive Differences (RMSSD). The ECG recording was divided into eleven 5-minutes timeframes corresponding to specific phases of the protocol (see Fig. 1): two baseline periods (within − 35 to -20 min), an anticipation phase (-7 to -2 min), two TSST phases (0 to + 5 and + 5 to + 10 min), and six early to late recovery phases (+ 12 to + 43 min). ECG data were visually inspected for artifacts using Kubios HRV Scientific software version 4.2.0 and corrected by two independent laboratory assistants. Artifacts (e.g., movement, ectopic beats) were removed together with the preceding beat. Timeframes with more than 15% corrected data were excluded from analyses. HR and HRV values were then averaged across each 5-minute timeframe.

#### Psychosocial variables

As potential moderators of the link between repression experience and stress responding, we measured resilience and perceived social support using the German versions of the Resilience Scale (RS-13^42^) and the Multidimensional Scale of Perceived Social Support (MSPSS^43^, as reported in a former publication^6^. Socioeconomic status (SES) was computed as an equally weighted composite score of income and education. In our preregistration, we additionally mentioned experience of transformation (time after German reunification) as a potential moderator. However, this exploratory measure resulted in predominantly missing data. Therefore it was excluded from all analyses.

For the exploratory secondary analyses only, we used measures originally reported in an earlier publication^6^, to assess anxiety (trait scale of State Trait Anxiety Inventory^40^, depression (Becks Depression Inventory^44^ and trauma symptoms (symptoms scale of the Harvard Trauma Questionnaire^45^.

### Data preparation and statistical analyses

All statistical analyses were conducted in R (version 4.5.0). Group differences in demographic variables (age, sex, SES) and PSS were examined using *t*-tests or chi-square tests. To reduce the impact of potential outliers, cortisol values were winsorized at ± 3 SD and log-transformed (natural log) due to non-normal distributions, as preregistered. Resilience, PSS and MSPSS were compiled as mean or sum scores, according to the respective author guidelines. To allow calculation of a valid sum score, for social support, single randomly missing items were imputed using multiple imputation with predictive mean matching (mice package, 50 iterations). For cortisol and STAI measures during the TSST, up to two missing values per participant were imputed the same way (see S.3 for detailed overview of all imputed data). As response scores, for cortisol, STAI, subjective stress, HR and HRV, areas under the curve with respect to ground (AUCg) and increase (AUCi) were calculated using all nine TSST measurement points. While the AUCg reflects the total stress output, AUCi provides a more direct measure of stress reactivity^46^. Although additional measures were preregistered, for clarity we focused on these two indices, which capture overall levels, reactivity, and recovery while utilizing all available data points. Additionally, cortisol responder rates were calculated, defined as an increase in cortisol level of ≥ 1.5 nmol/L from baseline levels following stress exposure^47^. For regression analyses, standardized regression coefficients (β) and (adjusted) *p*-values are reported. For significant effects, effect sizes are presented as partial η².

#### Stress response

We conducted separate linear regression models for each outcome (AUCg, AUCi) for cortisol, and our combined subjective stress measure to examine group differences (repression vs. control), controlling for age and sex due to their known influence on the stress response^48^. To correct for multiple testing, *p*-values of AUCg and AUCi were adjusted using false discovery rate (FDR) correction for each measure. Corrected p-values of 0.05 or lower were considered significant.

In a subsequent step, we tested whether interactions between group status and psychosocial factors (resilience, social support, and SES) were associated with stress reactivity outcomes. Accordingly, the three interaction terms (group × resilience, group × social support, and group × SES) were entered simultaneously into each regression model. Multicollinearity was evaluated and addressed by mean-centering all variables involved in the interaction terms. Although our preregistration proposed structural equation modeling to test a conceptual mediation, this approach was not feasible due to the high TSST dropout rates and an insufficient sample size. Therefore, we tested moderation models instead, examining whether psychosocial factors influenced the strength or presence of associations between repression and stress response outcomes. Assumptions underlying the regression analyses were inspected visually (see Supplementary Sections S.4).

#### Explorative analyses

As we modified our TSST setup after testing the first 16 participants (8 per group) following severe stress reactions in several repression group participants, we visually inspected differences between the participant groups of each testing protocol. Further, given the substantial difference in willingness and eligibility to participate in the TSST observed between control and repression groups, we conducted additional analyses comparing participants in the repression group who completed the TSST with those who did not and controls. These subgroups were compared in repression intensity, resilience, perceived social support and perceived stress. Additionally, we included differences in depressive symptoms, trait anxiety, and trauma-related symptoms (scales and original results reported in an earlier publication^6^. For each outcome, we fitted a one-way ANOVA with group (three levels) as a between-subjects factor. Significant main effects were followed up with Tukey HSD tests for multiple comparisons, reporting p-values.

## Results

**Fig. 2:**
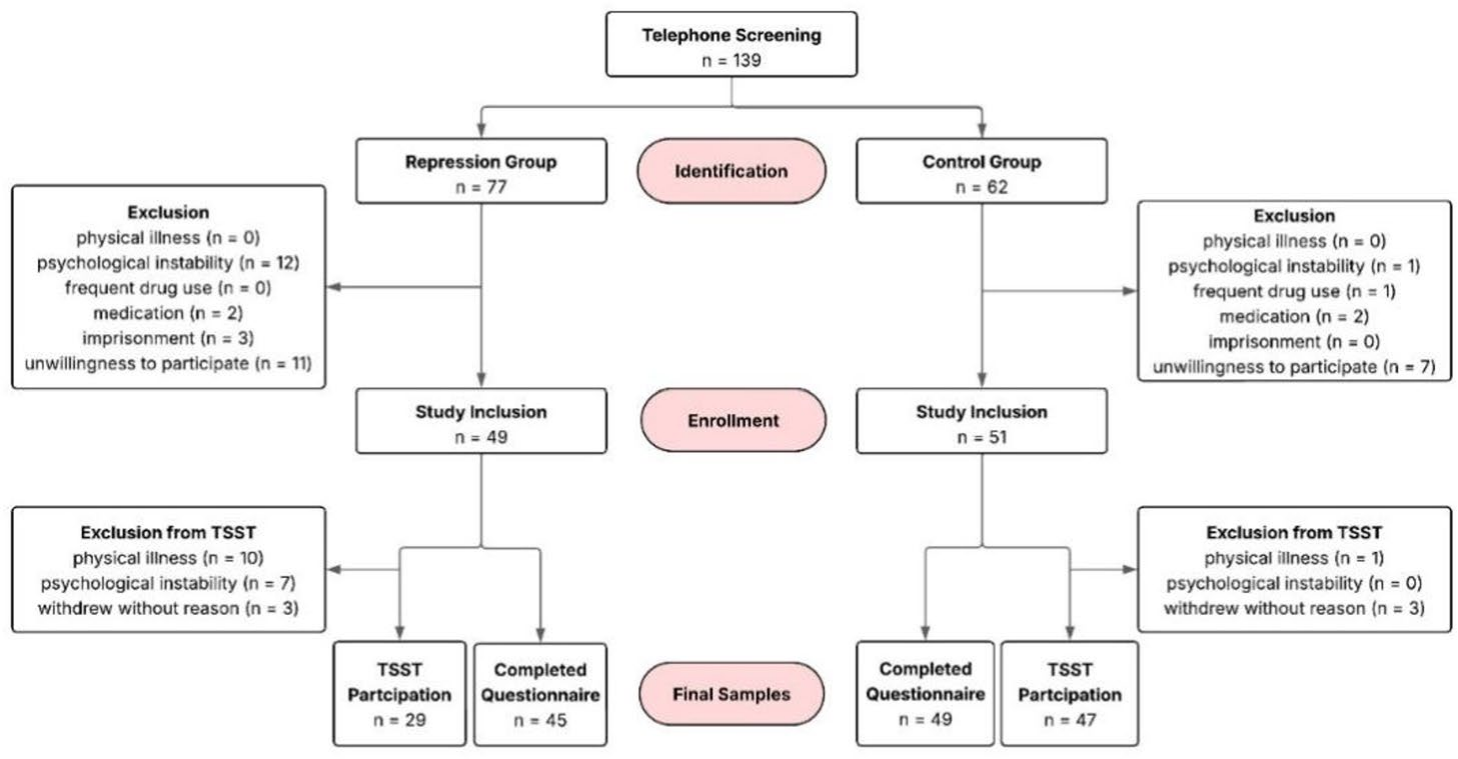
Flow diagram of inclusion process divided by repression and control group. This flowchart was created using lucidchart.com.

In total, we screened 139 individuals for eligibility using telephone interviews − 77 potential participants of the repression group, and 62 control individuals. Ultimately, 100 participants (49 in the repression group) were included in the study. Among those, twenty individuals in the repression group and four in the control group were excluded from or decided not to take part in the TSST (for details, see Fig. 2). There was a sample attrition rate of 40.8% for final HR and HRV analyses due to signal quality issues (*n* = 21 in the control group, *n* = 9 in the repression group). For cortisol and subjective stress data, the attrition rate was 1.3% (*n* = 1 in control group; see table S.5).

The samples of participants attending the TSST did not differ in age (*t*(67.07) = 0.21, *p* = .833), sex (*χ*^*2*^(1) = 0.62, *p* = .432), SES (*t*(52.33) = -0.01, *p* = .99), or chronic stress experience (PSS; (*t*(62.9) = -1.09, *p* = .281) between repression and control groups (for full descriptive information, see Table 1). As expected, repression intensity was significantly higher in the repression (*M* = 2.96), compared to the control group (*M* = 0.47; t(37.47) = -8.34, *p* < .0001).

**Table 1:** Demographic Information.

Variable	Control	Repression +TSST	Repression -TSST
Total N	47	29	20
Women	33 (70.2%)	17 (58.6%)	13 (65.0%)
Mean Age (SD)	65.28 (7.64)	64.93 (6.43)	67.30 (9.80)
Income			
< 1500€	5 (10.6%)	7 (24.1%)	6 (30.0%)
1500–4000€	32 (68.1%)	11 (37.9%)	8 (40.0%)
> 4000€	8 (17.0%)	9 (31.0%)	3 (15.0%)
No Information	2 (4.3%)	2 (6.9%)	3 (15.0%)
Religion			
Religious	10 (21.3%)	10 (34.5%)	9 (45.0%)
No Information	2 (4.3%)	2 (6.9%)	3 (15.0%)
Relationship status			
Single	5 (10.6%)	0 (0.0%)	4 (20.0%)
In relationship	5 (10.6%)	1 (3.4%)	0 (0.0%)
Married	26 (55.3%)	19 (65.5%)	8 (40.0%)
Divorced	5 (10.6%)	7 (24.1%)	5 (25.0%)
Widowed	4 (8.5%)	0 (0.0%)	0 (0.0%)
No information	2 (4.3%)	2 (6.9%)	3 (15.0%)
Children			
Yes, has Children	40 (85.1%)	24 (82.8%)	15 (75.0%)
No information	2 (4.3%)	2 (6.9%)	3 (15.0%)
Job status			
Working	19 (40.4%)	14 (48.3%)	5 (25.0%)
Jobless	1 (2.1%)	1 (3.4%)	0 (0.0%)
Pensioner	26 (55.3%)	14 (48.3%)	14 (70.0%)
Education			
< High school	16 (34.0%)	7 (24.1%)	6 (30.0%)
High School	2 (4.3%)	6 (20.7%)	1 (5.0%)
> High school	27 (57.4%)	14 (48.3%)	10 (50.0%)
No Information	2 (4.3%)	2 (6.9%)	3 (15.0%)

Note. Reported are absolute frequencies with percentages in brackets. For relationship status, multiple answers were possible. Full sample characteristics are reported in an earlier publication^6^.

Regression analyses revealed a significant group effect on the combined subjective stress score for overall stress (AUCg; *β* = 0.58, *p*_corr_ = 0.016, *η*^*2*^ = 0.08), with the repression group reporting higher stress, but not for stress reactivity (AUCi; *β* = 0.33, *p* = .129). Sex was a significant predictor of overall subjective stress (AUCg; *β* = − 0.55, *p*_corr_ = 0.030, *η*^*2*^ = 0.08) and stress reactivity (AUCi; *β* = − 0.49, *p*_corr_ = 0.033, *η*^*2*^ = 0.06), with men reporting lower stress levels than women. Age had no influence on overall subjective stress (AUCg; *β* = − 0.01, *p* = .528) or stress reactivity (AUCi; *β* = 0.03, *p* = .055) measurements. Please see supplement S.6–9 for separate STAI and subjective stress results, regression tables and sensitivity analyses.

Cortisol responder rates were 72% in the repression group and 65% in the control group, with no significant group difference (*χ*^*2*^ (1) = 0.16, *p* = .69). Linear regression models showed no significant effect of group on overall cortisol levels (AUCg; *β* = -22.33, *p* = .674) or stress reactivity (AUCi; *β* = 32.67, *p* = .546). Male participants showed higher overall cortisol (AUCg; *β* = 149.55, *p*_*corr*_ = 0.012, *η*^*2*^ = 0.09), but not cortisol reactivity (AUCi; *β* = 105.74, *p* = .073). Age had no effect on cortisol levels (AUCg: *β* = -2.19, *p* = .550; AUCi: *β* = − 0.76, *p* = .845.). See S.10 for regression tables.

**Fig. 3:**
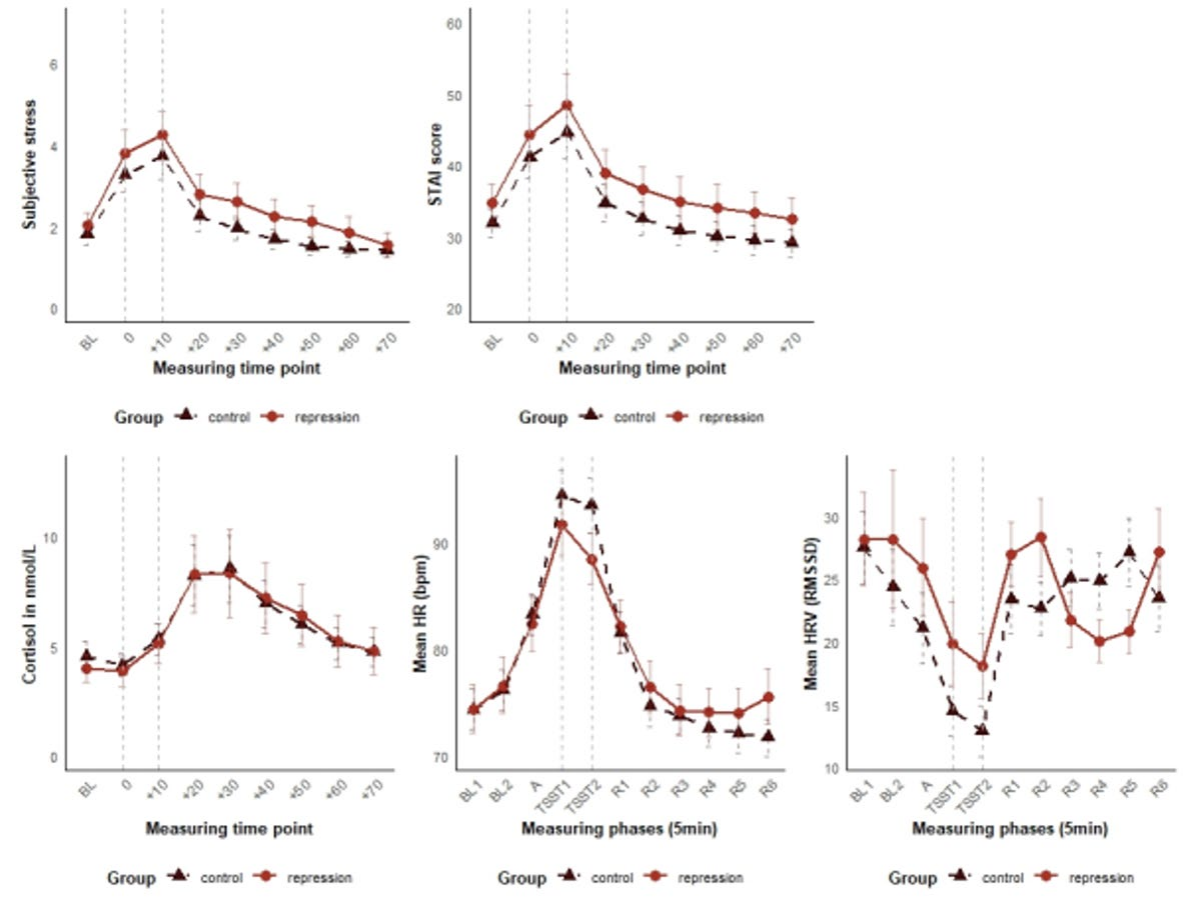
Stress levels during TSST laboratory testing and testing timeline. Mean levels (with 95% confidence intervals) of subjective stress (single-item Likert scale: “how stressed do you feel?”), STAI state, salivary cortisol (nmol/L), and mean heart rate (bpm) are shown across measurement time points during laboratory testing for the repression and control groups. Dashed vertical lines mark TSST onset and offset. In the HR and HRV panel, BL = baseline; A = Anticipation phase; R = recovery; TSST = Trier Social Stress Test.

The regression analyses revealed no significant group effect on overall HR (AUCg: *β* = 76.61, *p* = .723) or HR reactivity (AUCi: *β* = 0.75, *p* = .995) Sex and age had no influence on AUCg (sex: *β* = 31.89, *p* = .882; age: *β* = -23.89, *p* = .115) or AUCi (sex: *β =* -109.61, *p* = .326; age: *β* = 3.69, *p* = .631) heart rate measurements. Regarding HRV, the regression analyses revealed no significant group effect measured by AUCg (*β* = 93.46, *p* = .586) or AUCi (*β* = -13.3, *p* = .937). Again, sex and age had no influence on AUCg (sex: *β =* -32.22, *p* = .850; age: *β* = -4.03, *p* = .735) or AUCi (sex: *β =* 249.61, *p* = .137; age: *β =* -8.51, *p* = .464). Due to attrition rates of 40%, the HR and HRV results must be interpreted with caution (S.11). See Fig 3 for a depiction of subjective stress, cortisol, HR and HRV levels by group. . No moderation effects of social support, resilience, or SES were observed for overall stress or stress reactivity in subjective stress, cortisol, HR, or HRV (see Tables S.12) 3).

### Explorative analyses

We visually inspected graphical depictions of group differences in cortisol, subjective stress, HR and HRV responses to the TSST among the first 16 participants (8 participants per group), who completed the original TSST protocol prior to its modification due to severe distress in several repression group participants. The visual patterns observed in this subsample appeared to differ from the overall pattern and suggested higher cortisol levels and subjective stress responses in the repression group (See Fig. S.13). However, in this subsample, 50% of participants in the repression group were male, compared to only 14% in the control group. Therefore, it needs to be considered that the observed differences in cortisol reactivity reflect sex differences rather than effects of soft repression.

**Fig. 4:**
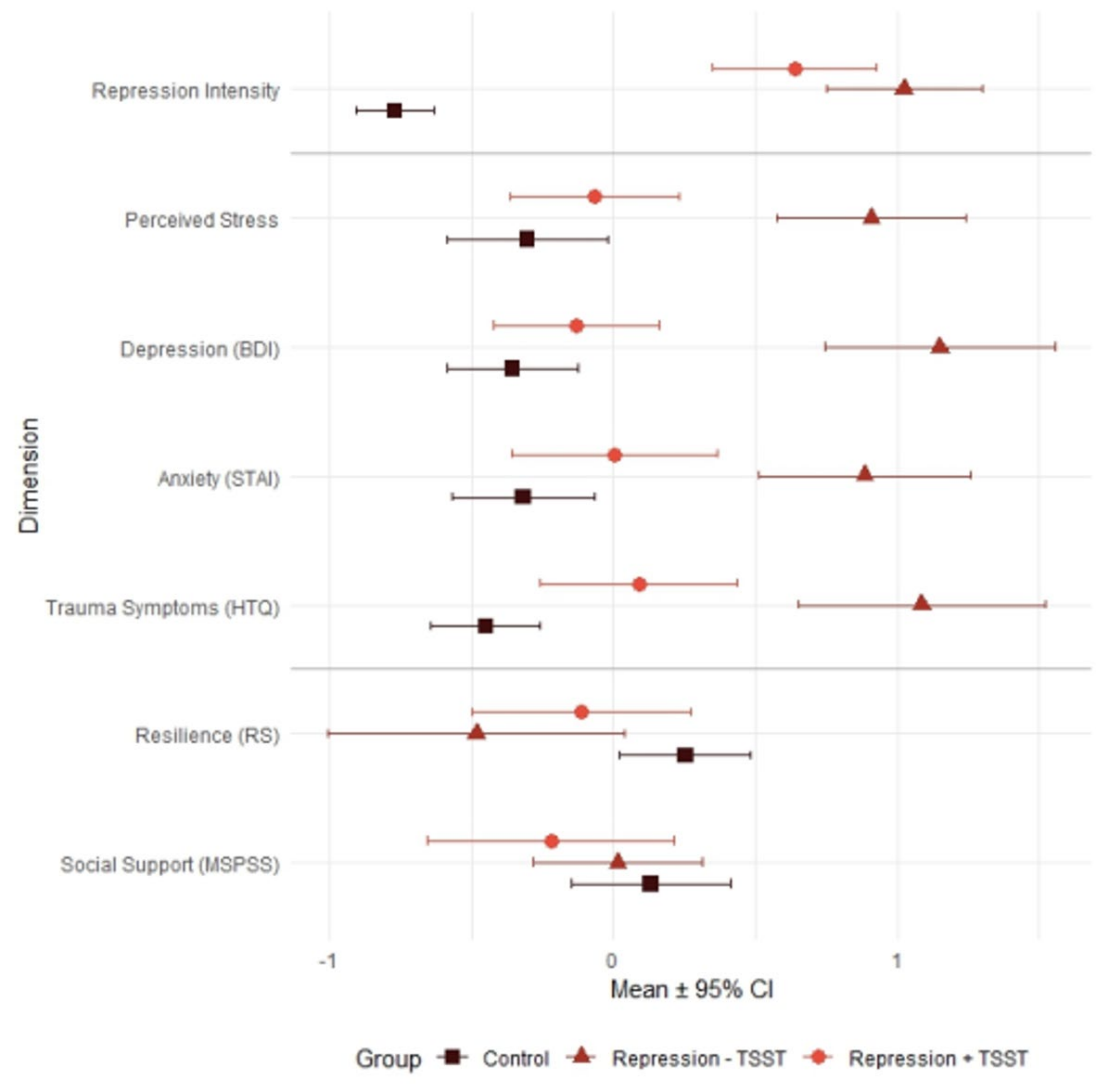
Forest plot of group differences in multiple dimensions. Plotted are mean differences (with 95% confidence intervals) for the repression group that participated in the TSST (TSST+), the repression group that did not participate in the TSST (TSST−), and the control group across several mean-centered dimensions. From top to bottom, the figure depicts repression intensity (sum-score composite of areas of life affected by repression), followed by distress-related variables: perceived stress (Perceived Stress Scale), depression (Beck Depression Inventory), anxiety (Trait scale of the State–Trait Anxiety Inventory), and trauma symptoms (symptom scale of the Harvard Trauma Questionnaire). Below the second horizontal divider, protective factors are shown: resilience (RS-13) and social support (Multidimensional Scale of Perceived Social Support).

One-way ANOVAs comparing the repression group conducting the TSST (TSST+), the repression group not conducting the TSST (TSST–), and the control group, followed by Tukey tests, revealed that both repression groups exhibited significantly higher repression intensity than the control group (both *p*s < 0.001), but did not differ from one another (*p* = .54; for an overview of group differences, see Fig. 4). With regard to distress-related outcomes, however, the TSST- group showed significantly higher levels of perceived stress, trauma symptoms, anxiety, and depression compared with both the TSST + and control groups (all ps < 0.01). No significant differences were observed between TSST + and control groups on any of the distress measures (all ps > 0.05). Concerning potential protective factors, the TSST- group differed significantly from the control group (*p* = .015), but not from the TSST+ group (*p* = .423) in resilience. TSST + and control groups showed no resilience differences (*p* = .199). No significant group differences were observed in perceived social support (MSPSS) among the three groups (all ps > 0.05). In summary, throughout all distress measures, TSST+ participants from the repression group were comparable to those from the control group, but differed from TSST- repression participants, suggesting higher vulnerability in those repression group individuals not included in the TSST. Please see S.14 for ANOVA tables of all measures.

## Discussion

The present preregistered study examined whether past exposure to soft political repression, experienced in terms of “Zersetzung” in the former GDR, is associated with long-term alterations in the psychobiological stress response. Compared to a matched control group, the repression group showed higher overall subjective stress during a standardized psychosocial laboratory paradigm, the Trier Social Stress Test (TSST^35^). Differences in subjective stress did not emerge when stress responses were measured in terms of acute reactivity. Also, no group differences emerged in cortisol levels, HR or HRV. Male (vs. female) participants reported lower subjective stress and stress reactivity while showing higher overall cortisol output. No moderation effects of SES, social support or resilience on subjective or cortisol stress levels were found. However, TSST attendance, which was voluntary and subject to additional exclusion criteria related to physical illness, was significantly lower in the repression group than in the control group. Exploratory analyses indicated that the repression subgroup not attending the TSST showed substantially higher levels of perceived stress, anxiety, depression, and trauma symptoms compared to both other (sub)groups, whereas repression group TSST completers were comparable to controls. Both repression subgroups did not differ in the reported intensity of repression experiences. No differences were observed in social support between the repression subgroups, and only small differences emerged in resilience, indicating that additional, unidentified factors may have contributed to the health trajectory after soft political repression experiences.

Contrary to our expectations, we did not find any differences in physiological stress responses between the repression and control groups. This may suggest that physiological stress systems were either not affected by soft repression, have recovered over the three decades since the repression experiences, or that the long-term mental health consequences of repression^6,49^ are operating through pathways other than physiological stress system activity. However, several methodological considerations warrant a closer examination of these findings. The pattern of participant exclusion itself provides valuable insight into the potential impact of repression. Particularly in the repression group, significant sample selection happened at multiple stages of the study. During the initial screening phase, considerably more repression group participants (vs. controls) were excluded from the study due to psychological instability, as defined by our study exclusion criteria of current psychopathology (12 repression vs. 1 control group participant), effectively excluding those who were potentially most clinically impacted by experiences of repression. Second, substantially more repression group participants were excluded from or declined participation in the TSST (20 vs. 4). Ten participants in the repression group were excluded due to physical illness, and another ten declined due to self-perceived psychological instability or personal reasons. Third, the TSST protocol itself was modified during data collection as the original protocol elicited pronounced distress in several repression-exposed individuals. Taken together, this successive selection and modification may explain why physiological stress responses did not differ between groups in the final analyses. The absence of group differences in stress reactivity may therefore reflect the predominance of particularly resilient repression group individuals in our final sample rather than the absence of long-term psychobiological consequences of repression experiences – especially since we did not observe comparable selection processes in the control group. This raises a broader methodological concern: Who are we actually studying in trauma and stress research?

Relatedly, it remains unclear why some individuals appear largely unaffected today, whereas others remain substantially burdened as exploratory findings in our study carefully suggest. One plausible explanation, requiring investigation in future research, concerns differences in stress appraisal, a key determinant of psychological and physiological stress responses^50^. In this population, appraisal may have been shaped by the context of repression. Individuals actively engaged in oppositional activities may have anticipated potential consequences and integrated repression into a coherent narrative of resistance (see e.g., sense of coherence^51^), potentially decreasing the key psychosocial stress components of uncontrollability, uncertainty and social devaluation^27^. In contrast, those targeted seemingly arbitrarily may have experienced greater uncertainty and self-doubt, fostering shame, a particularly potent emotion in trauma-related disorders^52^. This perspective is reflected in qualitative accounts (S.15). Several additional factors merit closer examination in future research, such as the very differential experiences of sociopolitical transformation in reunified Germany, age at repression onset or the duration of the repression experience. Taken together, our findings also tentatively convey the hopeful message of heterogeneity in vulnerability to soft repression and suggest the capacity for post-traumatic growth following adversity, that should be further explored in future work^53,54^.

### Limitations

Several study limitations should be considered in addition to the sampling bias discussed above. First, group allocation was inherently challenging. As all participants were exposed to the same sociopolitical system in the GDR, some degree of systemic repression was present across groups, resulting in an imperfect distinction between repression and control groups. In addition, repression experiences were highly heterogeneous, which reflects the complex nature of soft political repression but limits group homogeneity. Second, repression was assessed via retrospective self-report, which may be subject to memory bias or reinterpretation of past experiences. However, our measure of repression intensity showed a clear difference between repression and control groups, suggesting that group allocation was nevertheless successful. Regarding physiological data, a substantial proportion of HR/HRV data was affected by signal quality issues, resulting in limited interpretability due to reduced sample size. Similarly, our moderation models were most likely underpowered due to the large TSST dropout rates. Fourth, due to a sensitive population, we deviated considerably from the standard TSST protocol, which may limit comparability with prior studies. However, both experimental and control groups exhibited physiological and psychological stress responses, suggesting that less extreme stress paradigms may still be sufficient to elicit measurable psychobiological stress – albeit potentially insufficiently sensitive to detect nuanced group differences. Regarding our exploratory analyses, it should again be emphasized that these findings represent only initial indications and require further investigation in future research. Repression participants excluded from the TSST showed higher levels of physical illness, which may also explain their higher levels of psychological burden. At the same time, higher prevalence of physical illness is itself consistent with the hypothesis of long-term stress-related consequences^21^. As the groups were matched for age, sex, and origin, similar exclusion and non-participation rates would otherwise have been expected in the control group, which was not the case. Therefore, these findings, albeit exploratory and limited in interpretability, may point to underlying group differences and heterogeneity within the repression group.

## Conclusion

Soft political repression is not merely a phenomenon of the past, but remains highly relevant in the current global sociopolitical environment. The present study indicates that subgroups of affected individuals appear to have adapted, showing psychobiological outcomes comparable to those without experiences of political repression, while others experience mental and physical health challenges decades after exposure. Notably, the former group was more likely to participate in laboratory research. As demonstrated here, established laboratory approaches face challenges in capturing stress reactivity in highly vulnerable groups and may inadvertently exclude those most affected. This underscores the need to adapt research methodologies to better include these populations, for example through ecological momentary assessment in real-life settings using wearable devices or by examining polymorphic variance in stress-related genes^55^. Future research should test and extend our preliminary findings and identify the psychosocial and societal factors that differentiate successful long-term adaptation from persistent vulnerability to adverse health outcomes. Understanding such distinctions is essential not only for individualized healthcare approaches but also for advancing our understanding of resilience processes in sociopolitical contexts and for informing broader sociopolitical assessments - particularly in light of the ongoing global shift toward authoritarianism.

## Supplementary Information

Below is the link to the electronic supplementary material.

Supplementary Material 1

## Data Availability

The study protocol, informed consent forms, all R code and other relevant study material are provided on the OSF platform (osf.io/7xb8g). Deidentified participant data will be provided upon reasonable request.

## Code availability

This article was preregistered under: 10.17605/OSF.IO/S2C6P.
