# Supplementary material for: Long-term psychobiological stress responses following soft political repression
Source: Sci Rep. 2026 Aug 6;16:24306. doi: 10.1038/s41598-026-65049-8 (PMC13448652; doi:10.1038/s41598-026-65049-8)
Supplement: Supplementary file 1 [file 41598_2026_65049_MOESM1_ESM.docx]

**Supplementary Materials**

**Long-Term Psychobiological Stress Responses Following Soft Political Repression**

**S.1: Information about the TSST committee panel:**

Although original protocol recommends that the TSST committee consists of one male and one female confederate^1^, this was not always feasible for practical reasons. Consequently, our committee panels consisted of either one male and one female confederate or two female confederates. The distribution of committee compositions was similar across the repression and control groups. Moreover, a meta-analysis suggests that variations in TSST protocols, such as no visually present committee do not significantly influence salivary cortisol responses during the TSST^2^, making it unlikely that this variation affected our results.

1	Kirschbaum, C., Pirke, K. M. & Hellhammer, D. H. The 'Trier Social Stress Test'--a tool for investigating psychobiological stress responses in a laboratory setting. *Neuropsychobiology* **28**, 76-81, doi:10.1159/000119004 (1993).

2	Liu, J. J. W. *et al.* Sex differences in salivary cortisol reactivity to the Trier Social Stress Test (TSST): A meta-analysis. *Psychoneuroendocrinology* **82**, 26-37, doi:https://doi.org/10.1016/j.psyneuen.2017.04.007 (2017).

**S.2 Question on repression experiences**

**S.2.1 German original questionnaire version**

**S.2.2 English Translation:**

| *Below we list various forms of state abuse of power in the GDR (East Germany). Please indicate in each case whether you yourself, close relatives, extended family members, or friends were affected.* | | | | | |  |
| --- | --- | --- | --- | --- | --- | --- |
|  | Personally | Close relatives | Extended family | Friends | none | |
| 1. Political imprisonment |  |  |  |  |  | |
| 2. **Other politically motivated measures involving deprivation of liberty** (commitment to a clinic, placement in a GDR children’s home) |  |  |  |  |  | |
| 3. **Administrative measures** (interference with private property, assets, or custody and guardianship rights) |  |  |  |  |  | |
| 4. **Educational or professional disadvantages** (during education/training or in working life) |  |  |  |  |  | |
| 5. **Persecution by the State Security Service or other security organs** (surveillance, detentions, interrogation) |  |  |  |  |  | |
| 6. **Restrictions on personal freedom of opinion, religion, and movement** (punishment for expressing opinions, exclusion due to political or religious views, denial of permission to visit other countries) |  |  |  |  |  | |
| 7. Fear of being arrested, monitored, or having one’s career restricted |  |  |  |  |  | |

**S.2.3 Composition of “repression intensity” score**

The final repression intensity score was composited as a sum score of each “yes” Answer (=1) in question 2-7 in the “personally” column. Possible scores therefore ranged from 0 to 6.

**S.3 Imputed and Winsorized Data**

**S.3.1 Cortisol: Imputed Values**

| **Variable** | **Number of Imputed Values** |
| --- | --- |
| TSST3 | 1 |
| TSST4 | 3 |
| TSST5 | 2 |
| TSST6 | 1 |
| **Total** | 7 |

| **Variable** | **ID** | **Imputed Value** |
| --- | --- | --- |
| TSST3 | K02 | 10.15 |
| TSST4 | Z21 | 3.005 |
| TSST4 | Z29 | 6.485 |
| TSST4 | Z31 | 14.295 |
| TSST5 | Z29 | 5.165 |
| TSST5 | K41 | 5.105 |
| TSST6 | Z31 | 12.39 |

*Note:* *This table presents the single-value imputation performed for each participant across the nine cortisol measurement points. In total,* ***seven of 675*** *values were imputed.*

**S.3.2 Cortisol: Winsorized Values**

| **Variable** | **Number of Winsorized Values** |
| --- | --- |
| TSST1 | 2 |
| TSST2 | 1 |
| TSST3 | 1 |
| TSST8 | 2 |
| TSST9 | 1 |
| **Total** | 7 |

| **Variable** | **ID** | **BASE** | **Winsorized Value** |
| --- | --- | --- | --- |
| TSST1 | K10 | 11.27 | 10.68272 |
| TSST1 | K29 | 12.13 | 10.68272 |
| TSST2 | K10 | 10.64 | 9.51654 |
| TSST3 | K52 | 15.895 | 12.83536 |
| TSST8 | Z02 | 13.40 | 13.32516 |
| TSST8 | Z30 | 13.59 | 13.32516 |
| TSST9 | Z02 | 14.26 | 12.26048 |

*Note:* *This table presents the conducted winzorisations (3SD +/- mean) performed across all cortisol measurement points to control for outlier impact. In total,* ***seven of 675*** *values were winzorised.*

**S.3.3. STAI: Imputed Values, Single Items**

| **Variable** | **ID** | **Imputed Value** |
| --- | --- | --- |
| STAI_01_02 | K10 | 3 |
| STAI_01_07 | K10 | 2 |
| STAI_01_08 | K10 | 2 |
| STAI_01_09 | K27 | 2 |
| STAI_01_16 | K26 | 4 |
| STAI_01_18 | Z14 | 2 |
| STAI_02_02 | K48 | 3 |
| STAI_02_04 | K10 | 4 |
| STAI_02_10 | K41 | 3 |
| STAI_02_12 | Z39 | 1 |
| STAI_02_14 | K23 | 2 |
| STAI_03_02 | Z39 | 1 |
| STAI_04_03 | K52 | 3 |
| STAI_04_06 | K51 | 1 |
| STAI_04_10 | Z39 | 1 |
| STAI_05_02 | Z09 | 3 |
| STAI_05_06 | Z49 | 1 |
| STAI_05_07 | Z39 | 2 |
| STAI_05_16 | Z09 | 2 |
| STAI_06_06 | K23 | 1 |
| STAI_06_09 | K23 | 1 |
| STAI_06_14 | K20 | 1 |
| STAI_06_16 | K33 | 3 |
| STAI_06_16 | Z09 | 3 |
| STAI_06_19 | K02 | 3 |
| STAI_06_20 | K02 | 2 |
| STAI_08_06 | Z05 | 1 |
| STAI_08_07 | K14 | 2 |
| STAI_08_08 | K14 | 3 |
| STAI_08_12 | K51 | 2 |
| STAI_08_20 | K42 | 3 |
| STAI_09_11 | Z49 | 2 |

*Note:* *This table presents the single-value imputation performed for each participant across the nine STAI scales (20 items per scale). In total,* ***32 of 13,620*** *values were imputed. For this initial step, participants Z21 and Z31 were excluded from the STAI04 items, and Z31 was excluded from all STAI06 items, as they did not complete the respective questionnaires.*

**S.3.4 STAI: Imputed Values sumscores:**

| **Scale** | **ID** | **Imputed Value** |
| --- | --- | --- |
| STAI_04 | Z21 | 34 |
| STAI_04 | Z31 | 50 |
| STAI_06 | Z31 | 57 |

*Note:* *This table presents the imputation on a STAI sumscore level, performed for the two participants with entire missing questionnaires. In total****, three of 684*** *values were imputed.*

**S.3.5 Single Item subjective stress: Imputed Values**

| **Variable** | **Number of Imputed Values** |
| --- | --- |
| Ss_1 | 1 |
| Ss_2 | 2 |
| Ss_3 | 2 |
| Ss_4 | 3 |
| Ss_5 | 1 |
| Ss_6 | 1 |
| Ss_7 | 1 |
| Ss_8 | 1 |
| Ss_9 | 2 |
| **Total** | 14 |

| **Variable** | **ID** | **Imputed Value** |
| --- | --- | --- |
| ss_1 | Z02 | 3 |
| ss_2 | Z29 | 6 |
| ss_2 | Z30 | 5 |
| ss_3 | K15 | 2 |
| ss_3 | Z29 | 5 |
| ss_4 | Z21 | 2 |
| ss_4 | Z31 | 5 |
| ss_4 | Z49 | 1 |
| ss_5 | Z49 | 1 |
| ss_6 | Z31 | 5 |
| ss_7 | Z15 | 1 |
| ss_8 | Z49 | 1 |
| ss_9 | Z50 | 3 |
| ss_9 | 76 | 1 |

*Note:* *This table presents the single-value imputation performed for each participant across the nine single item subjective stress scales. In total,* ***14 of 675*** *values were imputed. For this step, participant K43 was excluded from imputation, as they had not completed four out of nine subjective stress items.*

**S.4 Visual Model Checks**

Depicted in the following S.3 graphs are visual model checks for each of the regression models, conducted within the package performance in R.

**S.4.1 Model cortisol AUCG (AUCg Cortisol ~ group + sex + age)**

**S.4.2. Model cortisol AUCi (AUCi Cortisol ~ group + sex + age)**

**S.4.3. Model AUCg cortisol with moderators (AUCg Cortisol ~ group * RS + group * MSPSS + group * SES)**

**S.4.4 Model AUCi cortisol with moderators (AUCi Cortisol ~ group * RS + group * MSPSS + group * SES)**

**S.4.5 Model subjective stress AUCg (AUCg subjective stress ~ group + sex + age)**

**S.4.6 Model subjective stress AUCi (AUCi subjective stress ~ group + sex + age)**

**S.4.7** **Model AUCg subjective stress with Moderators (AUCg subjective stress ~ group * RS + group * MSPSS + group * SES)**

**S.4.8 Model AUCi subjective stress with moderators (AUCi subjective stress ~ group * RS + group * MSPSS + group * SES)**

**S.5 Number of participants in each study part**

|  | **Cortisol** | **HR** | **HRV** | **SS** | **STAI** | **MSPSS** | **RS** | **SES** |
| --- | --- | --- | --- | --- | --- | --- | --- | --- |
| **repression** | 29 | 20 | 20 | 29 | 29 | 44 | 44 | 44 |
| **control** | 46 | 26 | 26 | 46 | 47 | 47 | 48 | 49 |

|  | **BDI** | **HTQ** | **STAI (trait)** | **Repression intensity** | **PSS** | **Sex** | **Age** |
| --- | --- | --- | --- | --- | --- | --- | --- |
| **repression** | 44 | 43 | 43 | 44 | 44 | 49 | 49 |
| **control** | 49 | 48 | 49 | 49 | 49 | 51 | 51 |

*Note:* *Final number of participants in each study part. Abbreviations: HR = Heat rate; HRV = Heart rate variability; SS = subjective stress single item; STAI = State Trait Anxiety Inventory during testing; MSPSS = Multidimensional Scale of Perceived Social Support; RS = Resilience-Scale13; SES = Approximate measure for socioeconomic status (equally weighted sumscore of education and income; BDI = Becks Depression Inventory; HTQ = Harvard Trauma Questionnaire (trauma symptoms scale); STAI(trait) = Trait scale of the State Trait Anxiety Inventory; Repression Intensity = Sumscore of number of repression measures experienced (see S.1); PSS = Perceived Stress Scale*

**S.6 Regression tables: Subjective stress composite score**

**S.6.1 Subjective stress (composite score) AUCg (AUCg subjective stress ~ group + sex + age)**

|  | **aucg_subsum** | | | |  |
| --- | --- | --- | --- | --- | --- |
| *Predictors* | *Estimates* | *std. Error* | *CI* | *p* | *p_corr_* |
| (Intercept) | -0.65 | 0.96 | -2.57 – 1.27 | 0.502 |  |
| group [repression] | 0.58 | 0.21 | 0.16 – 1.01 | **0.008** | **0.016** |
| sex [male] | -0.55 | 0.22 | -1.00 – -0.11 | **0.015** | **0.030** |
| age | 0.01 | 0.01 | -0.02 – 0.04 | 0.528 |  |
| Observations | 75 | | | |  |
| R^2^ / R^2^ adjusted | 0.147 / 0.111 | | | |  |

**S.6.2 Subjective stress (composite score) AUCi (AUCi subjective stress ~ group + sex + age)**

|  | **auci_subsum** | | | |  |  |
| --- | --- | --- | --- | --- | --- | --- |
| *Predictors* | *Estimates* | *std. Error* | *CI* | *p* | *p_corr_* |  |
| (Intercept) | -1.86 | 0.98 | -3.81 – 0.09 | 0.061 |  |  |
| group [repression] | 0.33 | 0.22 | -0.10 – 0.77 | 0.129 |  |  |
| sex [male] | -0.49 | 0.23 | -0.94 – -0.04 | **0.033** | **0.033** |  |
| age | 0.03 | 0.01 | -0.00 – 0.06 | 0.055 |  |  |
| Observations | 75 | | | |  |  |
| R^2^ / R^2^ adjusted | 0.118 / 0.081 | | | |  |  |

**S.7 Regression tables STAI**

**S.7.1 Subjective stress (STAI) AUCg (AUCg STAI ~ group + sex + age)**

|  | **aucg_stai** | | | |  |
| --- | --- | --- | --- | --- | --- |
| *Predictors* | *Estimates* | *std. Error* | *CI* | *p* | *p_corr_* |
| (Intercept) | 2614.50 | 708.29 | 1202.55 – 4026.44 | **<0.001** |  |
| group [repression] | 366.62 | 158.49 | 50.66 – 682.57 | **0.024** | **0.048** |
| sex [male] | -259.71 | 163.10 | -584.84 – 65.42 | 0.116 |  |
| age | 8.73 | 10.80 | -12.80 – 30.26 | 0.422 |  |
| Observations | 76 | | | |  |
| R^2^ / R^2^ adjusted | 0.094 / 0.056 | | | |  |

**S.7.2 Subjective stress (STAI) AUCi (AUCi STAI ~ group + sex + age)**

|  | **auci_stai** | | | |  |
| --- | --- | --- | --- | --- | --- |
| *Predictors* | *Estimates* | *std. Error* | *CI* | *p* | *p_corr_* |
| (Intercept) | -679.67 | 469.30 | -1615.19 – 255.86 | 0.152 |  |
| group [repression] | 109.29 | 105.02 | -100.05 – 318.64 | 0.301 |  |
| sex [male] | -208.45 | 108.07 | -423.87 – 6.98 | 0.058 |  |
| age | 15.02 | 7.16 | 0.75 – 29.28 | **0.039** | **0.078** |
| Observations | 76 | | | |  |
| R^2^ / R^2^ adjusted | 0.099 / 0.062 | | | |  |

**S.8 Regression tables subjective stress (single item)**

**S.8.1 Subjective stress (single item) AUCg (AUCg ss single item ~ group + sex + age)**

|  | **aucg_ss** | | | |  |
| --- | --- | --- | --- | --- | --- |
| *Predictors* | *Estimates* | *std. Error* | *CI* | *p* | *p_corr_* |
| (Intercept) | 174.97 | 83.25 | 8.98 – 340.96 | **0.039** |  |
| group [repression] | 51.29 | 18.48 | 14.45 – 88.13 | **0.007** | **0.014** |
| sex [male] | -58.69 | 19.14 | -96.85 – -20.53 | **0.003** | **0.006** |
| age | 0.65 | 1.27 | -1.89 – 3.18 | 0.613 |  |
| Observations | 75 | | | |  |
| R^2^ / R^2^ adjusted | 0.176 / 0.141 | | | |  |

**S.8.2 Subjective stress (single item) AUCg (AUCg ss single item ~ group + sex + age)**

|  | **auci_ss** | | | |  |
| --- | --- | --- | --- | --- | --- |
| *Predictors* | *Estimates* | *std. Error* | *CI* | *p* | *p_corr_* |
| (Intercept) | -66.46 | 68.09 | -202.23 – 69.30 | 0.332 |  |
| group [repression] | 27.33 | 15.11 | -2.81 – 57.46 | 0.075 |  |
| sex [male] | -33.15 | 15.65 | -64.37 – -1.94 | **0.038** | **0.038** |
| age | 1.75 | 1.04 | -0.32 – 3.83 | 0.097 |  |
| Observations | 75 | | | |  |
| R^2^ / R^2^ adjusted | 0.114 / 0.077 | | | |  |

**S.9 Sensitivity analyses combined subjective stress score without Z21 and Z31**

**S.9.1 Sensitivity Analysis AUCg**

|  | **aucg_subsum** | | | |  |
| --- | --- | --- | --- | --- | --- |
| *Predictors* | *Estimates* | *std. Error* | *CI* | *p* | *p_corr_* |
| (Intercept) | -0.41 | 0.95 | -2.31 – 1.49 | 0.668 |  |
| group [repression] | 0.50 | 0.21 | 0.07 – 0.92 | **0.022** | **0.044** |
| sex [male] | -0.50 | 0.22 | -0.93 – -0.06 | **0.027** | **0.027** |
| age | 0.01 | 0.01 | -0.02 – 0.03 | 0.712 |  |
| Observations | 73 | | | |  |
| R^2^ / R^2^ adjusted | 0.120 / 0.081 | | | |  |

**S.9.2 Sensitivity Analysis AUCi**

|  | **auci_subsum** | | | |  |  |
| --- | --- | --- | --- | --- | --- | --- |
| *Predictors* | *Estimates* | *std. Error* | *CI* | *p* |  | *p_corr_* |
| (Intercept) | -1.51 | 0.98 | -3.46 – 0.44 | 0.126 |  |  |
| group [repression] | 0.24 | 0.22 | -0.19 – 0.68 | 0.273 |  |  |
| sex [male] | -0.52 | 0.22 | -0.96 – -0.07 | **0.025** |  | **0.027** |
| age | 0.02 | 0.01 | -0.01 – 0.05 | 0.114 |  |  |
| Observations | 73 | | | |  |  |
| R^2^ / R^2^ adjusted | 0.103 / 0.064 | | | |  |  |

**S.10 Regression tables cortisol**

**S.10.1 Cortisol AUCg (AUCg cort ~ group + sex + age)**

|  | aucg_cort | | | |  |
| --- | --- | --- | --- | --- | --- |
| *Predictors* | *Estimates* | *std. Error* | *CI* | *p* | *p_corr_* |
| (Intercept) | 630.18 | 237.93 | 155.77 – 1104.60 | **0.010** |  |
| group [repression] | -22.33 | 52.87 | -127.74 – 83.08 | 0.674 |  |
| sex [male] | 149.55 | 54.47 | 40.94 – 258.17 | **0.008** | **0.012** |
| age | -2.19 | 3.64 | -9.45 – 5.08 | 0.550 |  |
| Observations | 75 | | | |  |
| R^2^ / R^2^ adjusted | 0.097 / 0.059 | | | |  |

**S.10.2 Cortisol AUCi (AUCi cort ~ group + sex + age)**

|  | auci_cort | | | |
| --- | --- | --- | --- | --- |
| *Predictors* | *Estimates* | *std. Error* | *CI* | *p* |
| (Intercept) | 140.32 | 253.60 | -365.35 – 645.99 | 0.582 |
| group [repression] | 32.67 | 56.35 | -79.68 – 145.03 | 0.564 |
| sex [male] | 105.74 | 58.06 | -10.02 – 221.51 | 0.073 |
| age | -0.76 | 3.88 | -8.50 – 6.98 | 0.845 |
| Observations | 75 | | | |
| R^2^ / R^2^ adjusted | 0.053 / 0.013 | | | |

**S.11 Regression Tables HR and HRV**

**S.11.1 Heart Rate AUCg (AUCg hr ~ group + sex + age)**

|  | **aucg_hr** | | | |
| --- | --- | --- | --- | --- |
| *Predictors* | *Estimates* | *std. Error* | *CI* | *p* |
| (Intercept) | 5438.93 | 961.86 | 3496.42 – 7381.44 | **<0.001** |
| group [repression] | 76.61 | 214.35 | -356.26 – 509.49 | 0.723 |
| sex [male] | 31.89 | 212.73 | -397.72 – 461.49 | 0.882 |
| age | -23.89 | 14.86 | -53.90 – 6.11 | 0.115 |
| Observations | 45 | | | |
| R^2^ / R^2^ adjusted | 0.061 / -0.008 | | | |

**S.11.2 Heart Rate AUCi (AUCi hr ~ group + sex + age)**

|  | **auci_hr** | | | |
| --- | --- | --- | --- | --- |
| *Predictors* | *Estimates* | *std. Error* | *CI* | *p* |
| (Intercept) | -301.91 | 493.80 | -1299.15 – 695.34 | 0.544 |
| group [repression] | 0.75 | 110.04 | -221.49 – 222.98 | 0.995 |
| sex [male] | -108.61 | 109.21 | -329.17 – 111.94 | 0.326 |
| age | 3.69 | 7.63 | -11.72 – 19.09 | 0.631 |
| Observations | 45 | | | |
| R^2^ / R^2^ adjusted | 0.028 / -0.043 | | | |

**S.11.3 Heart Rate Variability (RMSSD) AUCg (AUCg hrv ~ group + sex + age)**

|  | **aucg_rmssd** | | | |
| --- | --- | --- | --- | --- |
| *Predictors* | *Estimates* | *std. Error* | *CI* | *p* |
| (Intercept) | 1388.58 | 764.12 | -154.59 – 2931.75 | 0.076 |
| group [repression] | 93.46 | 170.28 | -250.43 – 437.35 | 0.586 |
| sex [male] | -32.22 | 168.99 | -373.51 – 309.07 | 0.850 |
| age | -4.03 | 11.80 | -27.86 – 19.81 | 0.735 |
| Observations | 45 | | | |
| R^2^ / R^2^ adjusted | 0.010 / -0.062 | | | |

**S.11.4 Heart Rate Variability (RMSSD) AUCi (AUCi hrv ~ group + sex + age)**

|  | **auci_rmssd** | | | |
| --- | --- | --- | --- | --- |
| *Predictors* | *Estimates* | *std. Error* | *CI* | *p* |
| (Intercept) | 40.90 | 744.63 | -1462.91 – 1544.71 | 0.956 |
| group [repression] | -13.30 | 165.94 | -348.42 – 321.82 | 0.937 |
| sex [male] | 249.61 | 164.68 | -82.98 – 582.19 | 0.137 |
| age | -8.51 | 11.50 | -31.73 – 14.72 | 0.464 |
| Observations | 45 | | | |
| R^2^ / R^2^ adjusted | 0.063 / -0.006 | | | |

**S.12 Moderation Analyses**

**S.12.1 Subjective stress (composite score) AUCg with moderators (AUCg subjective stress ~ group * RS + group * MSPSS + group * SES)**

|  | **aucg_subsum** | | | |  |
| --- | --- | --- | --- | --- | --- |
| *Predictors* | *Estimates* | *std. Error* | *CI* | *p* | *p_corr_* |
| (Intercept) | -0.12 | 0.14 | -0.39 – 0.16 | 0.403 |  |
| group [repression] | 0.31 | 0.22 | -0.13 – 0.74 | 0.159 |  |
| sumscore ses | -0.09 | 0.34 | -0.77 – 0.59 | 0.793 |  |
| RS mean | -0.63 | 0.30 | -1.23 – -0.04 | **0.038** | 0.076 |
| MSPSS score | -0.03 | 0.04 | -0.10 – 0.04 | 0.452 |  |
| group [repression] * sumscore ses | -0.02 | 0.50 | -1.03 – 0.99 | 0.966 |  |
| group [repression] * RS mean | -0.10 | 0.37 | -0.84 – 0.65 | 0.795 |  |
| group [repression] * MSPSS score | -0.00 | 0.05 | -0.10 – 0.09 | 0.920 |  |
| Observations | 68 | | | |  |
| R^2^ / R^2^ adjusted | 0.320 / 0.241 | | | |  |

**S.12.2 Subjective stress (composite score) AUCi with moderators (AUCi subjective stress ~ group * RS + group * MSPSS + group * SES)**

|  | **auci_subsum** | | | |
| --- | --- | --- | --- | --- |
| *Predictors* | *Estimates* | *std. Error* | *CI* | *p* |
| (Intercept) | -0.11 | 0.15 | -0.42 – 0.20 | 0.468 |
| group [repression] | 0.20 | 0.25 | -0.29 – 0.69 | 0.414 |
| sumscore ses | 0.38 | 0.38 | -0.38 – 1.15 | 0.319 |
| RS mean | 0.18 | 0.34 | -0.49 – 0.86 | 0.592 |
| MSPSS score | -0.03 | 0.04 | -0.11 – 0.05 | 0.429 |
| group [repression] * sumscore ses | -0.68 | 0.57 | -1.82 – 0.45 | 0.234 |
| group [repression] * RS mean | -0.69 | 0.42 | -1.54 – 0.15 | 0.105 |
| group [repression] * MSPSS score | 0.06 | 0.05 | -0.05 – 0.17 | 0.253 |
| Observations | 69 | | | |
| R^2^ / R^2^ adjusted | 0.116 / 0.014 | | | |

**S.12.3 Cortisol AUCg with moderators (AUCg cortisol ~ group * RS + group * MSPSS + group * SES)**

|  | aucg_cort | | | |
| --- | --- | --- | --- | --- |
| *Predictors* | *Estimates* | *std. Error* | *CI* | *p* |
| (Intercept) | 523.09 | 35.68 | 451.75 – 594.42 | **<0.001** |
| group [repression] | 19.25 | 57.30 | -95.33 – 133.84 | 0.738 |
| sumscore ses | 155.15 | 91.09 | -27.00 – 337.30 | 0.094 |
| RS mean | -52.72 | 72.13 | -196.95 – 91.50 | 0.468 |
| MSPSS score | 1.80 | 9.20 | -16.59 – 20.19 | 0.846 |
| group [repression] * sumscore ses | -105.12 | 134.32 | -373.72 – 163.47 | 0.437 |
| group [repression] * RS mean | 131.72 | 93.45 | -55.14 – 318.58 | 0.164 |
| group [repression] * MSPSS score | 1.48 | 12.54 | -23.60 – 26.56 | 0.906 |
| Observations | 69 | | | |
| R^2^ / R^2^ adjusted | 0.084 / -0.021 | | | |
| **S.12.4 Cortisol AUCi with moderators (AUCi cortisol ~ group * RS + group * MSPSS + group * SES)** | | | | |
|  | auci_cort | | | |
| *Predictors* | *Estimates* | *std. Error* | *CI* | *p* |
| (Intercept) | 97.52 | 37.37 | 22.80 – 172.24 | **0.011** |
| group [repression] | 75.46 | 60.02 | -44.56 – 195.48 | 0.213 |
| sumscore ses | 135.57 | 95.41 | -55.21 – 326.35 | 0.160 |
| RS mean | 45.86 | 75.54 | -105.20 – 196.92 | 0.546 |
| MSPSS score | 8.67 | 9.63 | -10.59 – 27.93 | 0.372 |
| group [repression] * sumscore ses | -152.45 | 140.69 | -433.77 – 128.88 | 0.283 |
| group [repression] * RS mean | -1.37 | 97.88 | -197.08 – 194.35 | 0.989 |
| group [repression] * MSPSS score | -6.62 | 13.13 | -32.89 – 19.64 | 0.616 |
| Observations | 69 | | | |
| R^2^ / R^2^ adjusted | 0.107 / 0.005 | | | |

**S.12.5 Heart Rate AUCg with moderators (AUCg hr ~ group * RS + group * MSPSS + group * SES)**

|  | **aucg_hr** | | | |  |
| --- | --- | --- | --- | --- | --- |
| *Predictors* | *Estimates* | *std. Error* | *CI* | *p* | *p_corr_* |
| (Intercept) | 3970.10 | 127.09 | 3712.11 – 4228.10 | **<0.001** |  |
| group [repression] | -139.32 | 189.33 | -523.68 – 245.04 | 0.467 |  |
| sumscore ses | -202.45 | 379.34 | -972.54 – 567.65 | 0.597 |  |
| RS mean | -45.47 | 313.40 | -681.70 – 590.76 | 0.885 |  |
| MSPSS score | -101.52 | 46.80 | -196.53 – -6.50 | **0.037** | **0.074** |
| group [repression] * sumscore ses | 87.07 | 507.20 | -942.60 – 1116.74 | 0.865 |  |
| group [repression] * RS mean | 272.63 | 380.18 | -499.18 – 1044.44 | 0.478 |  |
| group [repression] * MSPSS score | 59.93 | 53.08 | -47.82 – 167.68 | 0.267 |  |
| Observations | 43 | | | |  |
| R^2^ / R^2^ adjusted | 0.270 / 0.124 | | | |  |

**S.12.6 Heart Rate AUCi with moderators (AUCi hr ~ group * RS + group * MSPSS + group * SES)**

|  | **auci_hr** | | | |  | |  |
| --- | --- | --- | --- | --- | --- | --- | --- |
| *Predictors* | *Estimates* | *std. Error* | *CI* | *p* | *pcorr* | |  |
| (Intercept) | -155.39 | 70.86 | -299.24 – -11.53 | **0.035** |  |  |  |
| group [repression] | 62.68 | 105.57 | -151.63 – 277.00 | 0.556 |  |  |  |
| sumscore ses | 464.84 | 211.51 | 35.45 – 894.23 | **0.035** | 0.07 |  |  |
| RS mean | -131.14 | 174.74 | -485.89 – 223.61 | 0.458 |  |  |  |
| MSPSS score | 40.77 | 26.10 | -12.21 – 93.75 | 0.127 |  |  |  |
| group [repression] * sumscore ses | -474.79 | 282.81 | -1048.91 – 99.34 | 0.102 |  |  |  |
| group [repression] * RS mean | 190.08 | 211.98 | -240.27 – 620.43 | 0.376 |  |  |  |
| group [repression] * MSPSS score | -45.71 | 29.59 | -105.79 – 14.37 | 0.131 |  |  |  |
| Observations | 43 | | | |  | | |
| R^2^ / R^2^ adjusted | 0.239 / 0.087 | | | |  | |  |

**S.12.7 Heart Rate Variability (RMSSD) AUCg with moderators (AUCg hrv ~ group * RS + group * MSPSS + group * SES)**

|  | **aucg_rmssd** | | | |
| --- | --- | --- | --- | --- |
| *Predictors* | *Estimates* | *std. Error* | *CI* | *p* |
| (Intercept) | 1160.04 | 118.97 | 918.52 – 1401.56 | **<0.001** |
| group [repression] | 28.00 | 177.24 | -331.80 – 387.81 | 0.875 |
| sumscore ses | -109.49 | 355.11 | -830.39 – 611.42 | 0.760 |
| RS mean | -67.96 | 293.38 | -663.55 – 527.63 | 0.818 |
| MSPSS score | 8.16 | 43.81 | -80.79 – 97.10 | 0.853 |
| group [repression] * sumscore ses | -48.82 | 474.80 | -1012.72 – 915.07 | 0.919 |
| group [repression] * RS mean | 296.86 | 355.90 | -425.65 – 1019.37 | 0.410 |
| group [repression] * MSPSS score | -46.33 | 49.69 | -147.19 – 54.54 | 0.358 |
| Observations | 43 | | | |
| R^2^ / R^2^ adjusted | 0.140 / -0.032 | | | |

**S.12.8 Heart Rate AUCi with moderators (AUCi hrv ~ group * RS + group * MSPSS + group * SES)**

|  | **auci_rmssd** | | | |  |
| --- | --- | --- | --- | --- | --- |
| *Predictors* | *Estimates* | *std. Error* | *CI* | *p* | *pcorr* |
| (Intercept) | -444.19 | 105.15 | -657.65 – -230.73 | **<0.001** |  |
| group [repression] | 66.99 | 156.64 | -251.01 – 385.00 | 0.672 |  |
| sumscore ses | -153.67 | 313.85 | -790.82 – 483.48 | 0.627 |  |
| RS mean | 492.40 | 259.29 | -33.99 – 1018.80 | 0.066 |  |
| MSPSS score | 48.79 | 38.72 | -29.82 – 127.40 | 0.216 |  |
| group [repression] * sumscore ses | 560.27 | 419.64 | -291.65 – 1412.18 | 0.190 |  |
| group [repression] * RS mean | -699.18 | 314.55 | -1337.75 – -60.61 | **0.033** | 0.066 |
| group [repression] * MSPSS score | -10.11 | 43.91 | -99.26 – 79.04 | 0.819 |  |
| Observations | 43 | | | |  |
| R^2^ / R^2^ adjusted | 0.344 / 0.212 | | | |  |

**S.13 Subgroup analysis early TSST sample**

**Figure S.13 Stress levels in the subsample exposed to the original TSST protocol.**
***Note.*** *Mean levels (with 95% confidence intervals) of subjective stress (single-item Likert scale: “How stressed do you feel?”), state anxiety (STAI state), salivary cortisol (nmol/L), and mean heart rate (bpm) are shown across measurement time points during laboratory testing for the repression (*n *= 8) and control (*n *= 8) groups. Dashed vertical lines indicate TSST onset and offset. The figure is presented for exploratory and descriptive purposes only. Given the very small sample size, no statistical inferences are drawn. Heart rate and heart rate variability measures were omitted in this depiction due to the high attrition rate.*

**S.14 ANOVAs exploratory analyses subgroups**

| **ANOVA Results with FDR Adjustment** | | | | | | | |
| --- | --- | --- | --- | --- | --- | --- | --- |
| *Dependent_Variable* | *term* | *df* | *sumsq* | *meansq* | *statistic* | *p.value* | *p.adjusted* |
| Repression intensity | group | 1 | 169.545 | 169.545 | 137.948 | 0.000 | NA |
|  | Residuals | 87 | 106.927 | 1.229 | NA | NA | NA |
| BDI | group | 1 | 573.732 | 573.732 | 13.163 | 0.000 | 0.001 |
|  | Residuals | 87 | 3792.088 | 43.587 | NA | NA | NA |
| HTQ | group | 1 | 2.703 | 2.703 | 22.533 | 0.000 | 0.000 |
|  | Residuals | 85 | 10.196 | 0.120 | NA | NA | NA |
| STAI | group | 1 | 824.378 | 824.378 | 10.222 | 0.002 | 0.003 |
|  | Residuals | 86 | 6935.986 | 80.651 | NA | NA | NA |
| PSS | group | 1 | 2.654 | 2.654 | 9.105 | 0.003 | 0.005 |
|  | Residuals | 87 | 25.358 | 0.291 | NA | NA | NA |
| RS | group | 1 | 2.798 | 2.798 | 6.035 | 0.016 | NA |
|  | Residuals | 86 | 39.875 | 0.464 | NA | NA | NA |
| MSPSS | group | 1 | 28.257 | 28.257 | 1.493 | 0.225 | NA |
|  | Residuals | 85 | 1609.179 | 18.932 | NA | NA | NA |

**Note.** *ANOVA results of group differences between the repression group that participated in the TSST (TSST+), the repression group that did not participate in the TSST (TSST−), and the control group across several dimensions. Abbreviations: STAI = State Trait Anxiety Inventory (Trait scale); MSPSS = Multidimensional Scale of Perceived Social Support; RS = Resilience-Scale13; BDI = Becks Depression Inventory; HTQ = Harvard Trauma Questionnaire (trauma symptoms scale); STAI(trait) = Trait scale of the State Trait Anxiety Inventory; Repression Intensity = Sumscore of number of repression measures experienced (see S.1); PSS = Perceived Stress Scale*

**S.15 Quote on shame of study participant**

*“You somehow felt ashamed of something that, in fact, hadn’t really happened. Then you start to reflect and say, ‘Well, did something actually happen?’ So there was shame, I would say; I didn’t tell anyone about it either.” (Anonymous affected person #3, 25 August 2022).*
